# A hybrid computational model to predict chemotactic guidance of growth cones

**DOI:** 10.1038/srep11340

**Published:** 2015-06-18

**Authors:** Iolanda Morana Roccasalvo, Silvestro Micera, Pier Nicola Sergi

**Affiliations:** 1 The BioRobotics Institute, Scuola Superiore Sant’Anna, Pisa, Italy; 2 Bertarelli Foundation Chair in Translational NeuroEngineering Laboratory, Institute of Bioengineering, School of Engineering, Ecole Polytechnique Federale de Lausanne, Lausanne, Switzerland; 3 Center for Neuroprosthetics, Ecole Polytechnique Federale de Lausanne, Lausanne, Switzerland

## Abstract

The overall strategy used by growing axons to find their correct paths during the nervous system development is not yet completely understood. Indeed, some emergent and counterintuitive phenomena were recently described during axon pathfinding in presence of chemical gradients. Here, a novel computational model is presented together with its ability to reproduce both regular and counterintuitive axonal behaviours. In this model, the key role of intracellular calcium was phenomenologically modelled through a non standard Gierer-Meinhardt system, as a crucial factor influencing the growth cone behaviour both in regular and complex conditions. This model was able to explicitly reproduce neuritic paths accounting for the complex interplay between extracellular and intracellular environments, through the sensing capability of the growth cone. The reliability of this approach was proven by using quantitative metrics, numerically supporting the similarity between in silico and biological results in regular conditions (control and attraction). Finally, the model was able to qualitatively predict emergent and counterintuitive phenomena resulting from complex boundary conditions.

Axons establish highly specific connections to wire and develop the nervous system^1^. Pathfinding axons navigate through the body towards specific targets by sensing environmental characteristics^2^ and by following diffusible gradients of chemical cues^3^,^4^,^5^,^6^. This result is achieved by an extremely sensitive detector of chemical gradient: the growth cone^7^ (GC). The GC can sense diffusible gradients and move toward secretive targets^8^,^9^,^10^,^11^,^12^ through a chemotactic guidance process, which involves the amplification of external chemical signals through an internal transduction process^11^,^13^.

From a biological point of view, it is well known^14^ that calcium (Ca^2+^) is a key regulator of several important phenomena deeply involved in axonal guidance, as the promotion and inhibition of axonal outgrowth and growth cone turning^15^. Experimental correlations were found between directional increase of intracellular calcium concentration ([Ca^2+^]_i_) and biased protrusion of filopodia^16^,^17^,^18^,^19^ resulting in oriented outgrowth of axons^16^,^20^,^21^.

An increased level of [Ca^2+^]_i_ was found to be spread across the growth cone domain as well as clustered in micro-domains. These wide differences in gradient shape and magnitude were due to the complex interplay between Ca^2+^ homeostatic mechanisms and calcium influxes from membrane channels and calcium release from internal calcium stores^14^.

In addition, more complex, and unexpected axonal behaviours were described when extracellular calcium concentration ([Ca^2+^]_e_) changed, so the attractive/repulsive nature of guidance cues was related to both [Ca^2+^]_i_ and [Ca^2+^]_e_^21^. As a consequence, since intracellular and extracellular [Ca^2+^] influence each other, the GC membrane likely plays an important role in axonal steering, particularly through the dynamics of surface receptors^22^.

Since experiments show the presence of a chemotactic guidance of axons in crucial parts of body^23^,^24^ (e.g., brain, peripheral nerves), the investigation of mechanisms governing the directional outgrowth of axons is fundamental to improve our understanding of growth and regeneration processes (e.g., peripheral nerves, spinal root), and to foster the development of effective advanced strategies to tackle neural impairments and degenerative pathologies.

For this reason, computational models of axons have been often used in parallel to traditional experiments (directly performed on cells) to gather important and useful information^25^,^26^. Indeed, these models allowed the chemotactic outgrowth of axons to be investigated in depth for several boundary conditions^27^,^28^. They allowed also experimental evidences to be combined in different ways to investigate the logical strength of the cause-effect chain resulting in the final paths of axons.

More specifically, some models were implemented to investigate the intracellular signalling pathways underlying the chemotactic steering of GC. Through these pathways external stimuli are detected and translated into specific rearrangements of the GC cytoskeleton. Different subsets of signalling molecules were studied, such as the family of Rho GTPases Cdc42, Rac, and RhoA^29^,^30^ or the couple composed by calcium/calmodulin-dependent protein kinase II (CaMKII) and calcineurin (CaN)^31^,^32^, which are able to influence the levels of Ca^2+^ and cyclic adenosine monophosphate (cAMP). Recent experimental findings highlighted the role of the GC membrane, revealing an asymmetric redistribution of superficial receptors due to the presence of extracellular chemical gradients^22^. In addition, some works explored the positive feedback loop involved in the mechanisms of gradient amplification and cell polarization^33^,^34^. Further computational frameworks were implemented and allowed the step by step outgrowth of neurites to be reproduced^27^,^35^,^36^,^37^. These models were based on systems of differential equations, defining the outgrowth behaviour of GC, and able to account for extracellular conditions together with the GC response.

All these activities indicate the interest to obtain the knowledge of a set of “main rules” governing neuritic chemotaxis to replicate biological behaviours through simplified in silico models (i.e., via computer simulations). Nevertheless, the question on the nature of these rules is still open and interdisciplinary studies have been performed to link traditional and in silico experiments^37^,^38^.

Recently, a computational model was implemented^32^,^39^ to link experimental findings to in silico predictions. In particular, some intracellular signalling pathways, resulting in a combined action of calcium and cAMP, were considered to predict GC guidance decisions for a wide range of conditions. This model shows how the intracellular GC details are important to implement computational models able to make reliable previsions of “unexpected” and “unforeseen” behaviours (e.g., the switch from attractive to repulsive turning of axons in presence of attractive cues, as well as the switch from repulsive to attractive turning in presence of repulsive cues), which are relevant in neuroscience and in technological applications (e.g., regenerative neural interfaces^40^). These “unexpected” and “unforeseen” cellular behaviours are referred in the following as “counterintuitive behaviours”. Therefore, this work aimed at further proceeding with the use of computational models to predict axonal behaviours in different experimental conditions.

To this aim, a Java open-source software, CX3D^41^ (Institute of Neuroinformatics of ETH, Zurich), was used to implement a computational framework, that allowed developing neural networks to be modelled in a three-dimensional physical space. In addition, within this program, neurons were composed by discrete physical elements (e.g., spheres for somata, and cylinders for neurites) with tunable mechanical properties. Moreover, the outgrowth process of neurons was able to account for interactions among neighbouring objects and intracellular and extracellular diffusion of chemical cues. Although different biological processes (e.g., cell division and migration, extension of neuritic arbours and synaptic connection development) can be simulated within CX3D, neither a physical representation of GC nor an implementation of its behaviour are currently available. Therefore, in this work, the CX3D standard code was improved through the addiction of classes defining both GC morphology and filopodial protrusions. Similarly, the development of intracellular Ca^2+^ patterns was implemented within the GC, according to the presence of extracellular gradients of chemical cues. These internal fields were able to influence the choice of the leading filopodium guiding the multistep neuritic outgrowth through the GC advance.

In conclusion, the computational framework developed in this work allows the researchers to take advantage of several novel features. Indeed, realistic axonal trajectories were generated through an improved version of CX3D code, starting from a phenomenological approach to the activity of intracellular [Ca^2+^] within the GC domain. The growth cones were biomimetically implemented as monoconnected domains, and the whole evolution of internal chemical fields (i.e., activator/inhibitor) was implemented through a non standard Gierer-Meinhartd (GM) system^42^,^43^. Finally, the time variant process of axonal outgrowth was investigated and reproduced through multistep simulations, involving both the activity of growth cones and filopodia, while the suitability of the generated trajectories was tested through a quantitative comparison with literature experiments.

## Results

### In silico paths of outgrowing axons

An elliptic set of physical points (see Methods for further details) was used to implement the GC shape within CX3D^41^. Then, the current response of GC to the extracellular environment was obtained through a non standard GM system. This phenomenological approach accounted for the intracellular calcium dynamics, as resulting from interactions with extracellular cues and random fluctuations inside the GC domain. This coupled action was able to iteratively steer the filopodial elongation, and then the whole GC advance, in presence of attractive gradients or without chemical sources (control condition). Indeed, both of these behaviours were modelled to reproduce stereotyped biological behaviours of axons.

**Figure 1:**
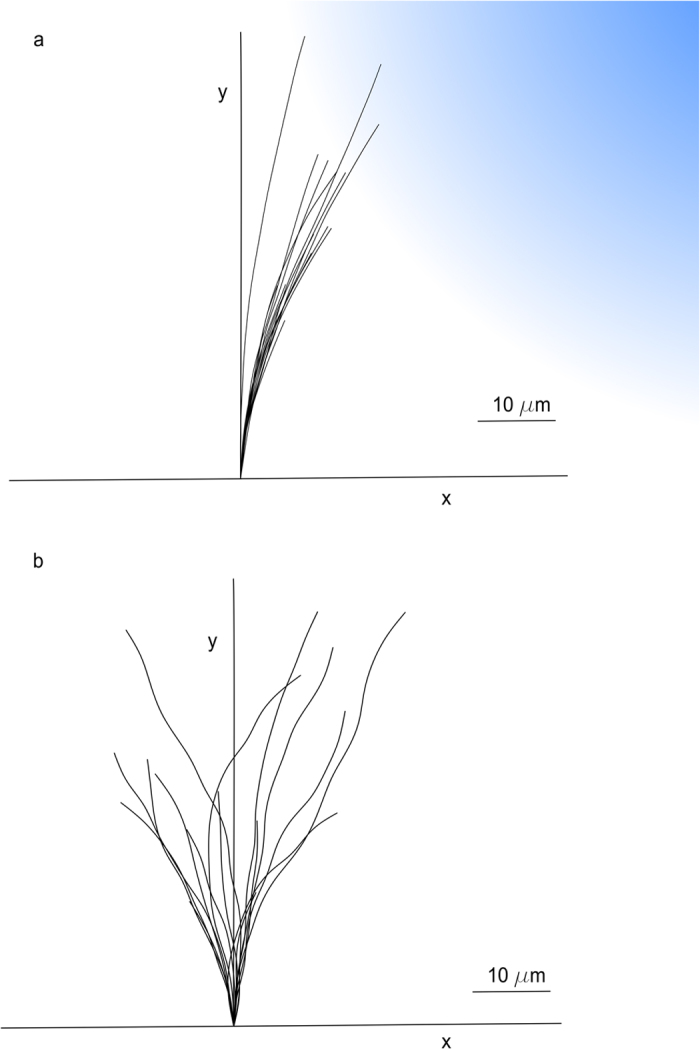
In silico axonal outgrowth for different extracellular conditions. The outgrowth trajectories of in silico axons (**a**) for an attractive gradient of netrin (gradation of blue colour) and (**b**) in control conditions (without gradient). In both cases, 16 traces of outgrowing axons are shown after a 1-h assay.

In Fig. 1a, the outgrowing trajectories of in silico growth cones (n = 16) are shown during a 1-h exposure to a netrin gradient. The chemical source was placed 100 μm away from the growth cone and at a 45 degree angle with respect to the initial growing direction, that was superimposed to the y-axis (the blue colour gradient indicated the chemical gradient direction). First, the exposure to an attractive gradient resulted only in small deflections of trajectories in direction of the source, while, at a later stage, axons widely turned since their outgrowth was more intensely guided by the shape of the extracellular gradient.

Similarly, in Fig. 1b, in silico growth cone traces (n = 16) are shown in control conditions. In this case, since no chemical gradients were present, the simulated axons did not show any preferential advance direction or biased deflections.

### Quantitative assessment of the axonal chemoattractive response

In silico turning angle and tortuosity values were measured and compared with experimental observations^44^ to quantitatively assess in silico performances in presence of a chemical gradient and in control conditions. In particular, the turning angle of axon was defined as the angle between the initial direction of growth and a conventional direction, given by the straight line linking the initial and the final position of the GC barycentre during the experimental assay.

Similarly, the tortuosity of the outgrowth traces was defined as the ratio of the curvilinear length of trajectory to the straight distance between its start and end points^37^.

**Figure 2:**
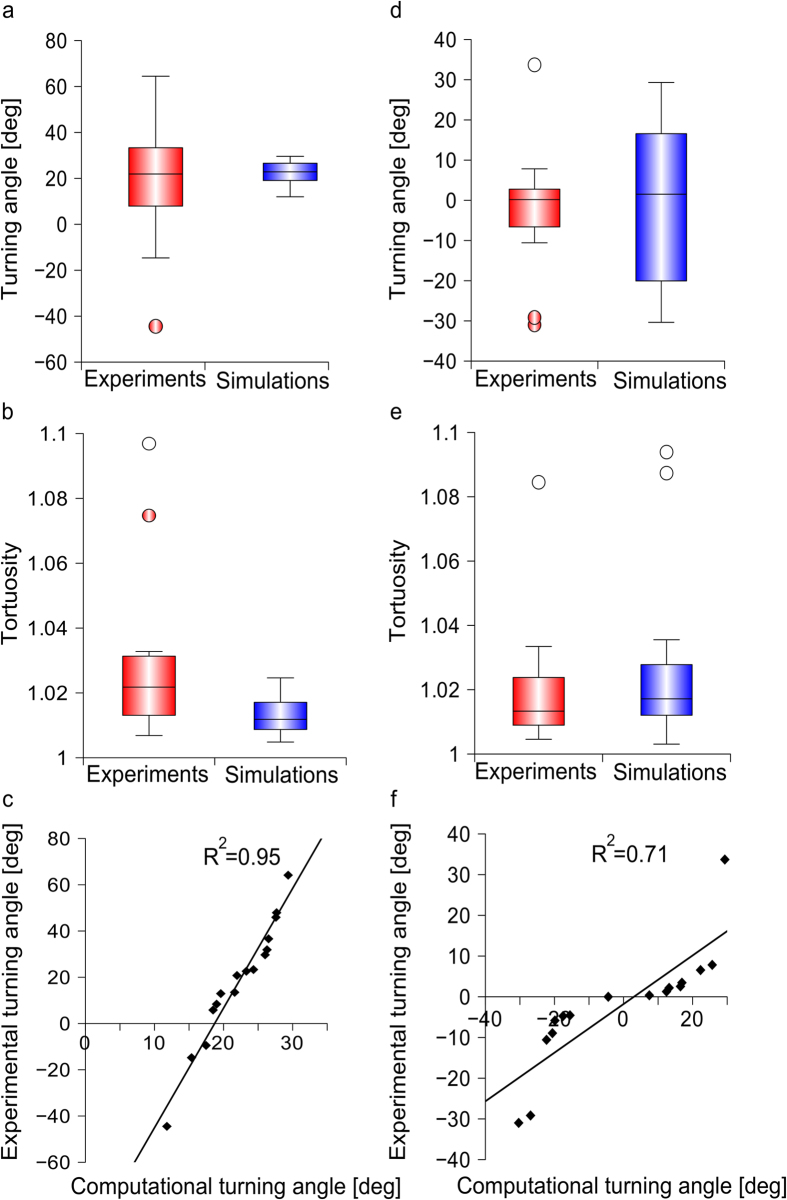
Assessment of in silico model performance for chemoattractive turning and control conditions. The explicit paths of in silico growth cones were measured in presence of an attractive netrin gradient and compared with the biological ones: (**a**) the difference between turning angles for experimental^44^ and simulated axons (n = 16 axons, for each set) was not statistically significant. (**b**) The difference between tortuosity of experimental^44^ (n = 10) and computational (n = 16) traces was not statistically significant. (**c**) Computational distribution of turning angles plotted versus experimental results^44^ (n = 16 samples for each case): a linear relationship was found (R^2^ = 0.95). Similarly, the explicit paths of in silico growth cones were measured in control conditions and compared with biological results: (**d**) turning angles for experiments^44^ and simulations (n = 16, for each set) were not significantly different. (**e**) Tortuosity values for experimental^44^ (n = 7) and simulated (n = 16) trajectories were not significantly different. (**f**) Computational distribution of turning angles plotted versus experimental results^44^ (n = 16 samples for each case): a linear relationship was found (R^2^ = 0.71).

In Fig. 2a, the distributions of experimental^44^ (n = 16) and simulated (n = 16) turning angles are shown after a 1-h exposure to a netrin gradient: the median values were similar (21.7° and 22.6°, respectively) as well as the whole distribution profiles. A Wilcoxon rank sum test was used to show that the difference between experimental and computational values was not statistically significant (p-value = 0.8091, 95% confidence level).

Then, the tortuosity of axonal paths was analysed (Fig. 2b) and the median value resulted in 1.022 for the experimental samples^44^ (n = 10), while for in silico axons the same value was 1.012 (n = 16). Also in this case, a Wilcoxon rank sum test showed that no significant differences were found between experiments and simulations (p-value = 0.06045, 95% confidence level). Finally, the correlation (R^2^ = 0.95) between experimental and in silico values of turning angles is shown in Fig. 2c.

### Quantitative assessment of axonal outgrowth in control conditions

In Fig. 2d, the turning angle distributions of experimental^44^ (n = 16) and computational (n = 16) samples were compared in control conditions (i.e., no extracellular gradients). The median values resulted, respectively, in 0.2° and 1.5°, and the range of variability was similar. Moreover, no statistically significant differences were found (Wilcoxon rank sum test, 95% confidence level, p-value = 0.6963).

In Fig. 2e, experimental^44^ (n = 7) and computational (n = 16) distributions of tortuosity are compared: the median values were respectively 1.013 and 1.017, and the range of variability was again similar. Furthermore, no statistically significant differences were found between in silico axons and experiments (Wilcoxon rank sum, 95% confidence level, p-value = 0.4921).

Finally, the correlation (R^2^ = 0.71) between in silico and experimental distributions of turning angles is shown in Fig. 2f.

### Dynamics of the intracellular activator

**Figure 3:**
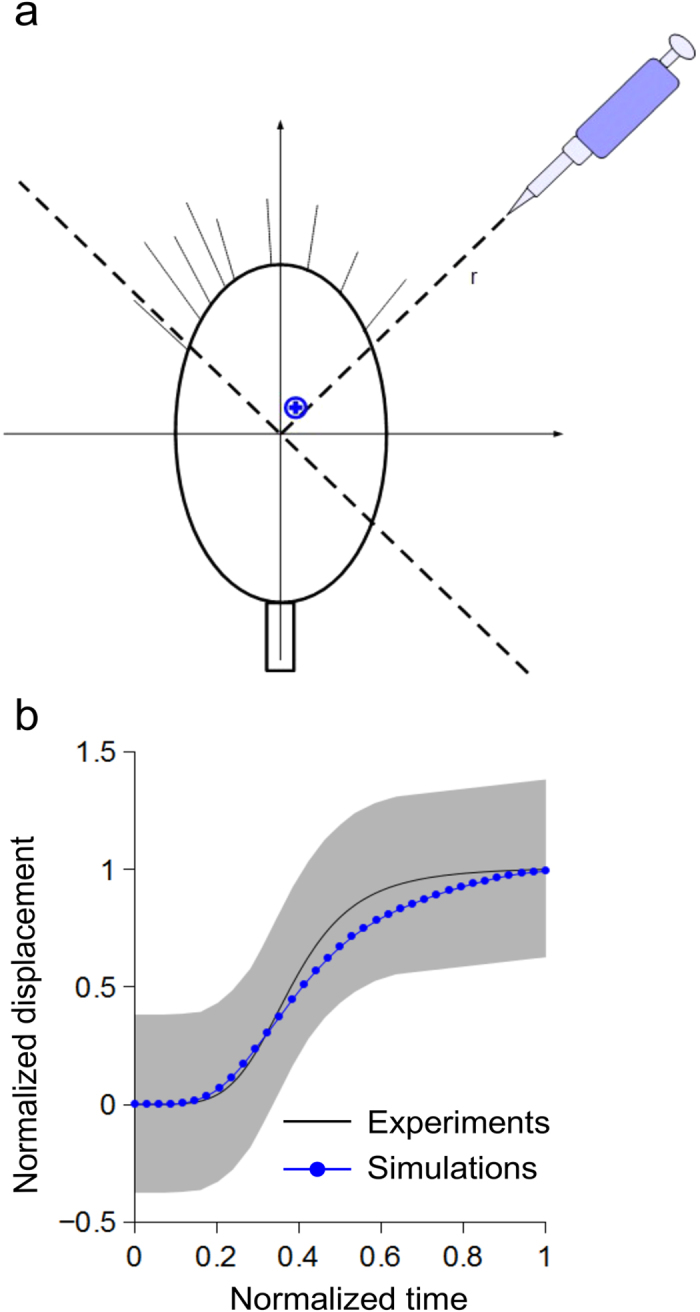
Dynamics of activator pattern development during gradient sensing. (**a**) In silico growth cones were implemented within the CX3D multiphysics framework, and the exposure to a chemoattractive gradient was simulated (the pipette indicated the gradient direction). In simulations, the displacement of the activator barycentre (blue cross) was observed along the bisector *r*. (**b**) The averaged behaviour (blue line, n = 20 simulations) of the activator barycentre was compared with the heuristic fit of GABA receptor dynamics (black line) reported by previous experiments^22^: the computational prediction was within the grey area, showing the range of biological variability.

The dynamics of activator (i.e., [Ca^2+^]_i_) pattern within the in silico growth cones was analysed. In Fig. 3a, a schematic representation of the pipette indicates the gradient direction, while the straight-line *r* identifies the bisector of the first and third quadrants. In Fig. 3b, the normalized average displacement of the activator barycentre along the straight-line *r* is reported for in silico growth cones (n = 20) and resulted in a sigmoid distribution. An initial short latency period was observed, when the activator distribution was symmetric and the barycentre displacement was close to zero. Then, since the activator barycentre moved toward the GC peripheral domain, its displacement gradually increased until it converged to a steady-state, and its position became stable. In Fig. 3b, the predicted curve (normalized) is compared with the experimental heuristic fit^22^, derived from the relocation of GABA (Gamma Amino Butyric Acid) receptors of 9 growth cones. In particular, the predicted curve and the experimental fit were similar and were within the bands of biological variability.

### Qualitative predictions of axonal counterintuitive behaviours

**Figure 4:**
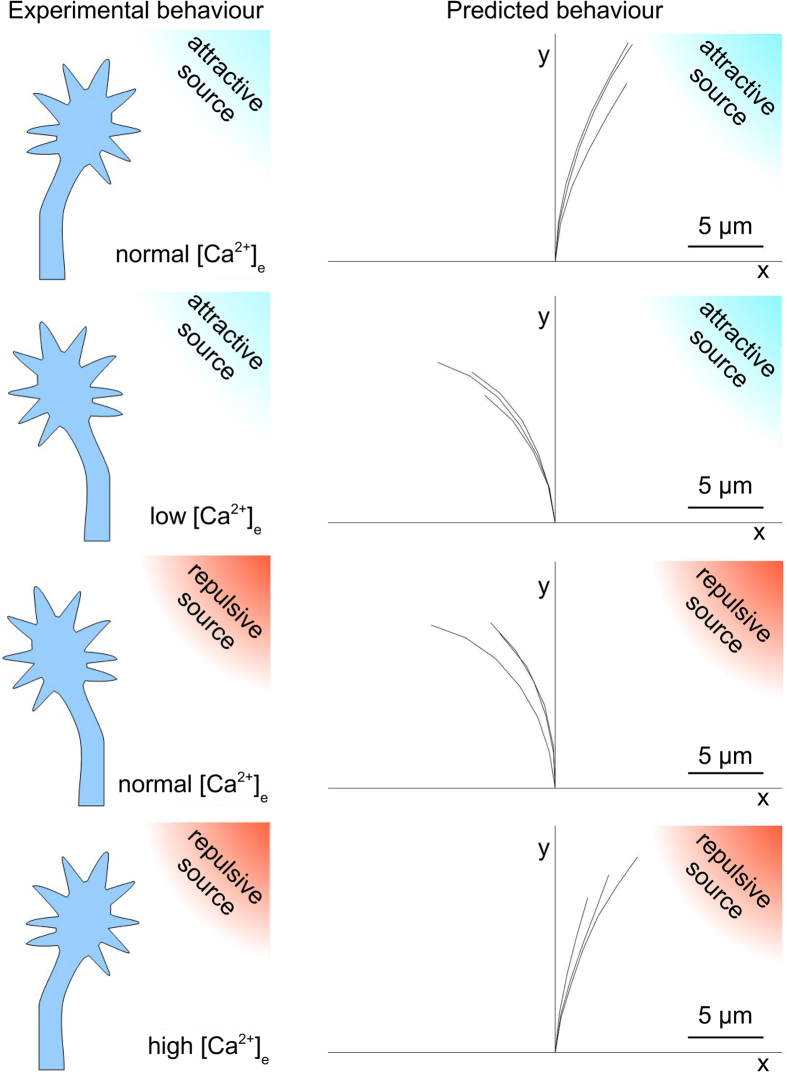
Counterintuitive predictions. Left side: cartoons of different biological behaviours of GC in presence of attractive (blue) and repulsive (red) cues for different extracellular concentration of calcium ([Ca^2+^]_e_). Upper panel: regular attraction towards an attractive cue. Upper-middle panel: counterintuitive behaviour, that is GC repulsion away from an attractive cue. Lower-middle panel: regular behaviour, that is GC repulsion away from a repulsive cue. Lower panel: counterintuitive behaviour, that is GC attraction towards a repulsive cue. Right side: qualitative predictions of the model of the biological behaviours. Upper panel: regular attraction towards an attractive cue. Upper-middle panel: counterintuitive behaviour, that is GC repulsion away from an attractive cue. Lower-middle panel: regular behaviour, that is GC repulsion away from a repulsive cue. Lower panel: counterintuitive behaviour, that is GC attraction towards a repulsive cue. The traces of the growth cone trajectories (n = 3, for each case) are shown.

In silico axons were implemented within the multiphysics framework of CX3D to account for the global effect of attractive/repulsive chemical sources coupled with the different levels of [Ca^2+^]_e_^39^. The capability of the model to reproduce counterintuitive responses of biological axons was then tested. In Fig. 4, the observed biological behaviours are schematically represented on the left side of the panel, while in silico traces (n = 3, for each case) are shown on the right side.

For each simulation, an extracellular chemical source was implemented 100 μm away from the growth cone and at a 45 degree angle with respect to the direction of y-axis (the nature of the sources is indicated through the coloured gradient: respectively red and blue for repulsive and attractive sources).

When [Ca^2+^]_e_ was about 0.9 mM (i.e., normal culture conditions^32^), the extracellular attractive gradient induced an attractive steering of the growth cone towards the source (upper panel).

Similarly, when the extracellular calcium level decreased (upper-middle panel), in silico growth cones turned away from the source, showing the influence of the decreasing level of [Ca^2+^]_e_ on the switch from attraction to repulsion, according to the biological behaviour.

Moreover, when an extracellular repulsive gradient was implemented together with a normal level of [Ca^2+^]_e_ (0.9 mM), axons underwent a repulsive turning and migrated away from the chemical source (lower-middle panel). However, an increment of the extracellular calcium level was sufficient to elicit an attractive turning on the side of growth cone facing the chemical source (lower panel).

## Discussion

### A multistep time-variant algorithm

**Figure 5:**
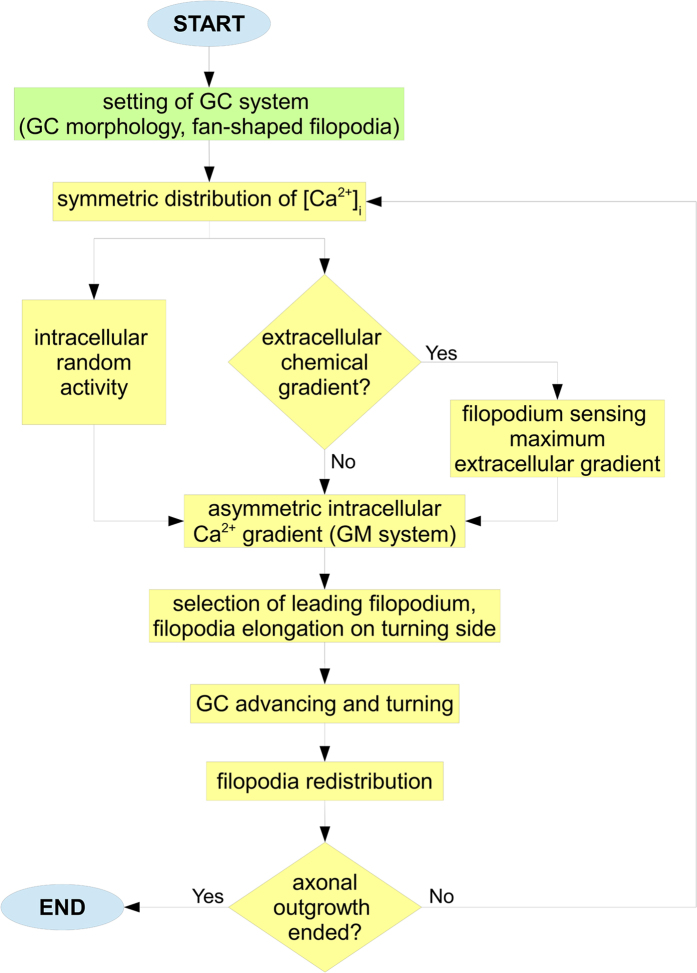
A multistep outgrowth behaviour. At the beginning of simulations, the biological system was set (green block). In particular, the GC morphology was defined through an elliptic set of physical nodes, extending filopodia with a fan-shaped disposition. Then, the GC advance was iteratively implemented. For each outgrowth step (yellow loop), starting from a symmetric distribution, the intracellular Ca^2+^ dynamics of GC evolved (GM system) according to the interaction between the sensing of extracellular cues and random fluctuations of internal GC activity. Specifically, the exposure to a chemical source elicited an asymmetry of [Ca^2+^]_i_ on the up-gradient side of GC. This phenomenon was mediated by the activation of a filopodium sensing the maximum gradient. On the contrary, in absence of extracellular gradients, [Ca^2+^]_i_ spots randomly appeared on GC boundary, influenced by the intracellular random activity of GC. In both cases, the appearance of a [Ca^2+^]_i_ peak allowed the GC to identify the turning side through the elongation of filopodium angularly closest to the peak (i.e., the leading one). Finally, GC moved and randomly redistributed its filopodia on boundary. Then, the sequence of sensing and movement was repeated for all outgrowth steps.

The flow diagram of the algorithm used to implement the in silico simulations is shown in Fig. 5. First, the biological system was set within CX3D (green block) for each simulation. Then, an elliptic shape for the GC was implemented through a set of physical points, together with a fan of emerging filopodia, radially extending to probe the extracellular environment. Therefore, since biological paths of outgrowing axons show several “decision points” (i.e., points where the GC seems to decide the right turning direction), the in silico process of outgrowth was implemented through a multistep algorithm, and the number of iterative steps was set according to experimental observations^44^.

For each step (yellow loop), starting from a symmetric distribution, the Ca^2+^ dynamics evolved according to a non standard Gierer-Meinhardt system^42^,^43^, where two chemicals, activator (e.g., intracellular Ca^2+^) and inhibitor (e.g., a Ca^2+^ antagonist), diffused, interacted and converged to a steady-state pattern of reciprocal concentration. In addition, the exposure to an extracellular chemoattractive source induced a polarized asymmetry of [Ca^2+^]_i_ on the up-gradient side of GC.

This process was mediated by the activation of the filopodium sensing the maximum gradient, as in Fig. 5. However, in control conditions (i.e., no external chemical gradients), the spots of Ca^2+^ (areas of high concentration) were still influenced by the random intracellular activity of GC, so spots randomly appeared on its boundary. In both cases, the position of the main [Ca^2+^]_i_ peak directly influenced the choice of the GC side to turn. This process was implemented through the selection of the leading filopodium, which was the filopodium angularly closest to the peak.

The elongation of the leading filopodium was also able to define a turning angle for the whole GC structure. Once the GC turned following this direction, filopodia were symmetrically redistributed on its boundary. So, the sequence of GC sensing and movement was iteratively repeated for all steps, steering the in silico axon advance toward the chemical source (chemical active environment) or according to a random walk (control case).

### A hybrid approach

The internal biochemical activity of the growth cone was implemented through a non standard GM^42^,^43^ system to follow a hybrid phenomenological approach. Indeed, from a side, researchers aim at modelling the exact set of biochemical reactions^32^, which take place inside the growth cone in response to chemical cues. From the other side, the extreme complexity of the intracellular signalling cascades, together with the presence of several calcium influxes coming from different sources (e.g., superficial channels and internal stores), makes very difficult to reliably model the biochemical activity of the growth cone through explicit equations. In addition, currently, it is not completely clear^39^ how different influxes of calcium could interplay within the domain of the growth cone. As a consequence, in this work the relationship between morphological evolution of axons (outgrowth paths) and the internal biochemical activity of the growth cone was kept by using a GM-like system, which was a further improvement of the Turing approach^45^. This approach allows the chemical nature of the phenomenon to be described through a phenomenological description. Indeed, only a couple of chemical compounds, deriving from complex intracellular signalling cascades, was necessary without the need of further molecular details. The only requirement was the existence of chemical compounds playing the roles of “activator” and “inhibitor” (e.g., antagonist to the increase without bounds of the first one). Moreover, both chemicals could be calcium compounds. Probably, these roles may be currently assigned to CaMKII and CaN^39^. A phenomenological description of the biochemical activity of the GC was chosen to keep the whole model more general and to allow its dynamic part (e.g., the axon outgrowth) to be developed independently from the chemical derivation of the calcium gradient within the growth cone domain. In this way, the dynamic part of the model could be potentially coupled with several different chemical descriptions to test their capability to produce correct paths of outgrowing axons. This kind of phenomenological description was capable to produce spots of activator (and consequently gradients of calcium ions) very similar to those experimentally described^1^,^15^ in presence of chemical cues. Nevertheless, the phenomenological nature of the model implied that numerical values of the GM system were not immediately referred to physical characteristics of chemical compounds. However, the use of this hybrid approach allowed the implementation of complex behaviours with only two equations as well as the integrity of the growth cone domain to be preserved for each step of outgrowth.

### Sensitivity and concentration

In this model, the growth cone was assumed to sense the external environment through a directional clustering of filopodial receptors along the direction of the chemical source^27^. For the sake of simplicity, for each step, this cluster was assumed to be located at the base of the actual filopodium sensing the maximum gradient. Moreover, an “amplification” function was used to account for the growth cone capability to sense external chemical compounds in a wide range of concentrations^1^. The growth cone sensitivity was, then, implemented as non trivially related to the extracellular concentration of chemicals. In addition, the location of the maximum of this sensitivity function indirectly accounted for the centre of the cluster of filopodial receptors.

### Deterministic and stochastic phenomena were coupled in simulations

For each step of growth, the initial levels of activator and inhibitor on the growth cone domain underwent random fluctuations to reproduce natural conditions^15^. Similarly, the distribution of filopodia on the growth cone boundary was taken from a uniform distribution, to account for their natural variability. Then, the position of the maximum of the sensitivity function, together with initial random fluctuations of morphogens, was capable to stochastically influence the outcome of the GM system, which was responsive to the extracellular environment for each step of simulation. This is crucial, because the “classic” GM system is strictly deterministic (once fixed all parameters) and then it is unsuitable to be used “as it is” to model the iterative outgrowth of axons.

### Explicit paths of outgrowing axons in chemical attractive and control environment

The model was capable to provide the explicit paths of axons starting from the interplay between extracellular and intracellular environments. For each step, the actual position of the growth cone was calculated according to the position of the leading filopodium, that, in its turn, depended on the interaction between outer and internal chemical fields, as well as on stochastic processes (see previous paragraph). Nevertheless, the question arose about how to honestly compare “in silico” results with biological traces. In this work, a quantitative definition of “similarity” was used: in silico and biological traces were defined “similar”, whether the median values of turning angle and tortuosity of simulated traces were near to the experimental median values. The suitability of the whole procedure was, then, tested by comparing in silico and biological traces through these quantitative metrics. Moreover, since the GC was assumed to display stereotyped behaviours, a further comparison with the control case (no chemical cues) was performed. In other words, the same simulations were capable to provide in silico paths respectively similar (according to the previous definition) to experimental traces both in presence and absence of chemical cues.

### Counterintuitive axonal behaviours

Counterintuitive behaviours of outgrowing axons were experimentally described^21^,^32^, and the global influence of chemical cues was found to simultaneously depend on their intrinsic nature (attractive, repulsive) as well as on the calcium concentration in the extracellular environment. As usual, attractive boundary conditions led to the turning of the growth cone towards the chemical source. Attractive conditions were experimentally obtained when attractive chemicals, a steep internal gradient of calcium and (at least) a normal extracellular concentration of calcium were coupled. Similarly, an attractive behaviour was obtained when a repulsive cue was coupled with a high ionic concentration of calcium within the extracellular environment. In the same way, repulsive conditions led to the turning of the growth cone away from the source. These conditions were obtained when a repulsive chemical cue was coupled with a shallow intracellular gradient and a moderate extracellular concentration of calcium, as well as when an attractive source was coupled with a low concentration of extracellular calcium. In silico simulations were capable to qualitatively model the behaviour of growth cone for all these cases. Indeed, as shown in Fig. 4, attractive and repulsive behaviours were implemented by using all the previous described combinations of chemicals, internal gradients and extracellular calcium concentrations. In silico growth cones were implemented to choose the leading filopodium accounting for, at the same time, the internal gradient of activator, the nature of the chemical cue, and the extracellular concentration of calcium. In case of attractive conditions, the leading filopodium was chosen on the up-gradient side of the growth cone according to literature^27^,^39^, while in case of repulsive conditions the leading filopodium was on the down-gradient side^39^. This phenomenological description was chosen to reproduce the global result of a complex series of biochemical and biophysical processes. In case of attractive boundary conditions, this global effect phenomenologically accounted for the steep increase of the calcium gradient and the consequent asymmetric production of β_1_-integrins within the growth cone domain, their local cytosolic saturation, the increase of exocytosis (e.g., mediated by VAMP2 (vesicle-associated membrane protein 2), Sytx (syntaxin) and TI-VAMP (neurotoxin-insensitive VAMP))^39^, the consequent asymmetric diffusion and precipitation of β_1_-integrins into focal adhesion complexes^46^, and, finally, the asymmetric local increase of stress due to neuritic shortening^47^. Similarly, in case of repulsive boundary conditions, the global in silico effect phenomenologically accounted for the shallow increase of the calcium gradient with a moderate production of β_1_-integrins into the growth cone domain, the activation of endocytosis and transmigration of β_1_-integrins towards the down-gradient side of the growth cone^39^, the antilateral concentration and precipitation of β_1_-integrins leading to adhesion complexes, the consequent antilateral increase of the local stress due to neuritic shortening.

### Coupling between chemical receptors and calcium dynamics

Receptors on the growth cone surface were found to dynamically move and react to the extracellular environment. In particular, the presence of a GABA source was found to induce the asymmetric redistribution of GABA receptors towards the source, as well as an enhancement in the asymmetry of intracellular calcium concentration in spinal neurons of rat^22^. Intriguingly, as shown in Fig. 3b, after normalization, the experimental dynamics of GABA receptors was similar to the in silico dynamics of the barycentre of the activator concentration. Since specific receptors are linked to Ca^2+^/K^+^ channels, the dynamics of receptors indirectly shows the dynamics of channels^48^. As a consequence, regardless to the cellular mechanism that allows the receptors to be directionally clustered, the qualitative likeness between the two normalized curves may indicate a not negligible role of the L-type channel influxes of Ca^2+^ on the whole calcium dynamics and distribution on the growth cone domain.

## Conclusions

The computational approach to the modelling of outgrowing axons presented in this work can be used as a versatile tool to support and integrate biological investigations. Indeed, in silico modelling of axons allows the main rules governing chemotaxis to be implemented, with a chosen level of accuracy, at the growth cone level. As a consequence, the strength of the logic chain connecting all experimental findings can be tested for several permutations of extracellular and intracellular conditions to result in biologically suitable paths of axons. Moreover, the minimum set of necessary rules to implement the natural phenomenon can be investigated and the relevance of each of them can be tested. Furthermore, a possible logic connection between the superficial dynamics of receptors and the intracellular activity of the growth cone could be computationally explored with several different boundary conditions. Finally, the computational framework provided in this work was implemented by using an open Java software, and freely offers to the whole scientific community the following characteristics:

### Generality

A non standard GM system was used to reproduce the biochemical nature of the intracellular processes. Although it involved two biochemical compounds, it was not essentially dependent on their nature. Therefore, this system could be applied to different chemical pathways resulting in different final chemical compounds, capable to affect the attractive or repulsive turning response of the GC.

### Domain integrity and possible time-variance of GC shape

The non standard GM system was simultaneously applied to the whole domain of the GC and resulted in an internal gradient of calcium ions. This made simulations more biomimetic. Although, for the sake of simplicity, in this work the domain was kept constant through the time (i.e., the growth cone had an elliptic geometry), it could have, in general, a time-variant shape. Indeed, the number of nodes defining the GC domain, and where the GM system was implemented, could be changed to account for exocytosis due to attraction (increase of the node number), or endocytosis due to repulsion (decrease of the node number).

### Intracellular/extracellular communication

The in silico GCs were sensible to the external environment through a GM algorithm (deterministic) computed within random boundary conditions resulting in a non standard GM system of equations. This approach accounted for the influence of the extracellular environment, and resulted in a field of intracellular calcium simultaneously dependent on intracellular and extracellular conditions.

### Growth cone adaptation and extracellular calcium concentration

The in silico GCs were capable to discern the different, but coupled, actions of extracellular chemical gradient and intracellular sensitivity. This approach was useful to simulate the capability of the growth cone to sense chemicals in a wide range of concentrations (adaptation).

### Sensing/desensing cycles

The GM-like system was iteratively implemented over the whole GC domain for every new displacement of the GC. This approach was suitable to account for the changes in boundary conditions and implement the sensing/desensing cycles characteristic of real GCs.

### Biological noise

Natural GCs undergo several changes (e.g., shape, number of protruding filopodia, intracellular concentration of calcium ions, etc.). In silico GCs accounted for random effects through stochastic fluctuations on initial concentration of intracellular calcium and a changing homogeneous distribution of filopodia, mimicking the natural disposition of filopodia.

### Internal chemical fields and explicit paths

This model, starting from the internal biochemical fields, allowed the trajectories of GCs to be explicitly derived and traced. The displacement of the barycentre of the GC area was considered to trace paths.

### Quantitative comparison between in silico and biological paths

In silico traces were compared to biological paths through quantitative indexes (turning angle, tortuosity) and for both control and attractive conditions. This approach avoided a simply heuristic and visual comparison.

### Counterintuitive behaviours of GC

In silico GCs were capable to reproduce some counterintuitive biological behaviours. In particular, they were able to reproduce the attraction/repulsion switch depending on the extracellular concentration of calcium as well as on both the steepness of the internal calcium gradient and the nature of the chemical source.

### Openness and further extensibility

In silico paths of outgrowing axons were iteratively implemented. For each step, the growth cone accounted for the interactions between intracellular and extracellular environments to decide the correct turning procedure. These interactions could be extended to time-variant phenomena belonging to a multiphysics domain. In particular, several factors may be changed or added (e.g., chemical sources, different topographies of substrates^49^, electromagnetic fields as light or laser^50^, etc.), as well as time-variant and coupled phenomena.

## Methods

### Evolution of intracellular calcium patterns

The evolution of the calcium gradient within the growth cone domain was modelled through a GM system^42^,^43^, where a self-enhancement mechanism, accomplished by an “activator” morphogen, was balanced by an antagonistic reaction involving an “inhibitor”. In the model, the term “activator” was used to account for the calcium activity, while the role of “inhibitor” was conferred to any chemical compound involved in calcium homeostasis and whose activation was necessary to regulate and maintain the intracellular calcium levels within the biological ranges^51^. As a consequence, the evolution of intracellular calcium gradients was described by the following system of partial differential equations^43^:


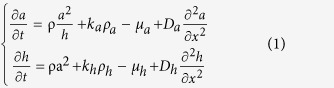


where the calcium concentration (*a*) in a growth cone microdomain (i.e., each physical node used to discretise the growth cone domain) at time t depended on the contribution of four processes: diffusion, degradation, production, and finally interaction with an antagonist morphogen, whose concentration was *h*. Similarly, ρ was a production coefficient; μ_*a*_ and μ_*h*_ were the decay coefficients. D_*a*_ and D_*h*_ were the diffusion coefficients; ρ_*a*_ and ρ_*h*_ were the basal production coefficients, and k_*a*_ and k_*h*_ were numerical coefficients.

**Figure 6:**
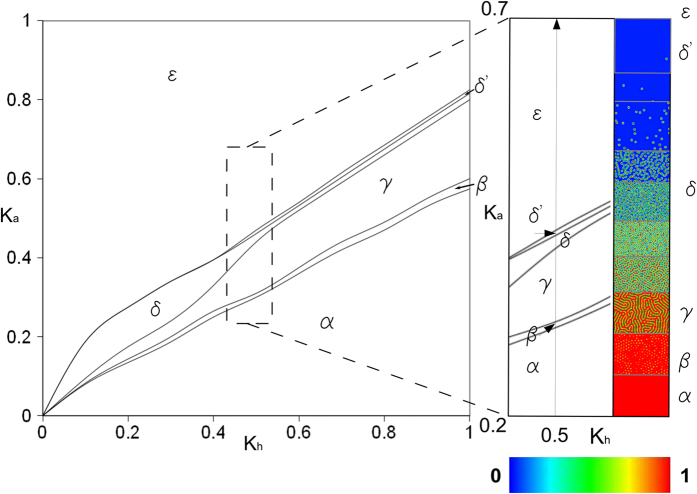
A versatile Turing like model. A wide range of behaviours were modelled by using the GM system. Only two phenomenological equations were capable to produce different activator patterns (identified with different Greek letters: α,β,γ,δ,δ',ε) when the coefficients k_a_ and k_h_, respectively related to the basal production of activator (**a**) and inhibitor (h), varied between 0 and 1. On the right side of the Figure, a small part of the phase plane was magnified to show the bidimensional projections of the activator pattern when k_a_ = 0.5 and k_h_ = [0.2,0.7].

The system of Equations (1) allowed a phenomenological representation of a wide series of intracellular patterns to be implemented with a change of constant parameters. This behaviour is shown in Fig. 6 for changes of k_a_ and k_h_ values, which were related to the basal production of activator and inhibitor in the range [0, 1] (left). Different patterns were identified with different Greek letters (i.e., α,β,γ,δ,δ',ε) and the magnification of a part of plane (i.e., near to k_a_ = 0.5 and for k_h_ = [0.2,0.7]) is shown in Fig. 6 (right) to visualize the activator pattern associated to different regions of the left phase plane.

**Figure 7:**
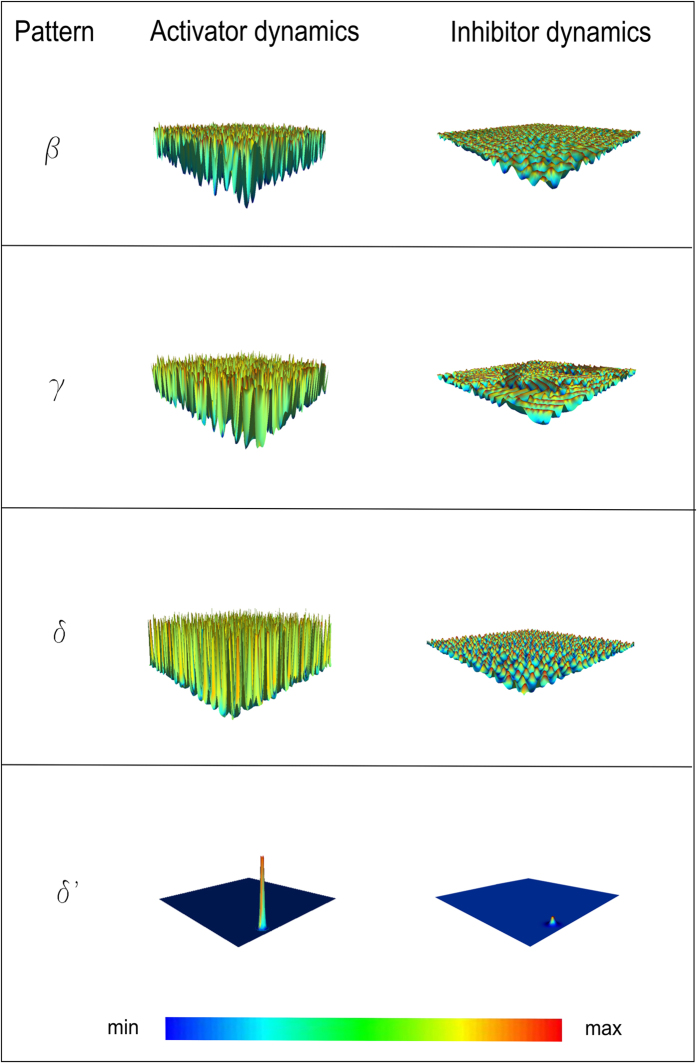
Activator and inhibitor patterns. For each three-dimensional pattern of activator (modular surfaces: β,γ,δ,δ'), the coupled three-dimensional pattern of inhibitor is shown. Both of these patterns were simultaneously present within the domain of simulation.

In addition, in Fig. 7 for each three-dimensional pattern of activator (left column) the coupled pattern of inhibitor is shown (right column). These patterns were simultaneously implemented within the growth cone domain during in silico trials.

In our simulations, for each in silico growth cone microdomain, the value of *a* accounted for the contribution of three terms:


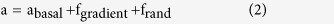


Here, *a*_basal_ was the basal concentration of calcium, and *f*_*gradient*_ was the variation induced by the sensing of the extracellular source. Finally, *f*_*rand*_ was a random term due to the growth cone intracellular activity^15^. This formulation allowed two different growth cone behaviours to be modelled: chemotactic response to an extracellular gradient and random walk advance in control conditions.

More specifically, *f*_*rand*_ was a Delta dirac function whose value was different from zero in a node randomly chosen on the growth cone boundary. Furthermore, since experiments support the growth cone capability to perceive very low gradients of chemical compounds, the variation of the activator concentration, due to the extracellular source, was modelled through an amplification function. In particular,


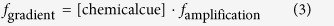


where


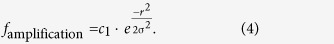


and *r* represented the distance of each microdomain from a maximum sensitivity point on the growth cone surface, that was identified at the base of filopodium extending in direction of the maximum gradient.

### Convergence of calcium pattern

The Shannon information entropy was used to assess the minimum number of iterations for the GM process to allow the evolution of activator within growth cone domain to converge toward a stable pattern.

To this aim, the standard in silico growth cone was implemented as a set of physical nodes, forming a two-dimensional elliptic body, where each node was initialized with random values of *a* and *h* taken from a uniform distribution. The activator concentration *a* was used to evaluate the entropy, that was defined as:


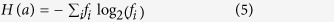


where *f*_*i*_ was the probability that *a* belonged to the i-esime range of concentration and f_*i*_ log_2_(f_*i*_ ) = 0 if f_*i*_ = 0. The value of entropy was then plotted for a high number of iterations (i.e., 20000 time steps) to reflect the evolution of the activator pattern in time, allowing the steady-state of pattern to be assessed.

### Computational model of axonal outgrowth guided by chemotaxis

**Figure 8:**
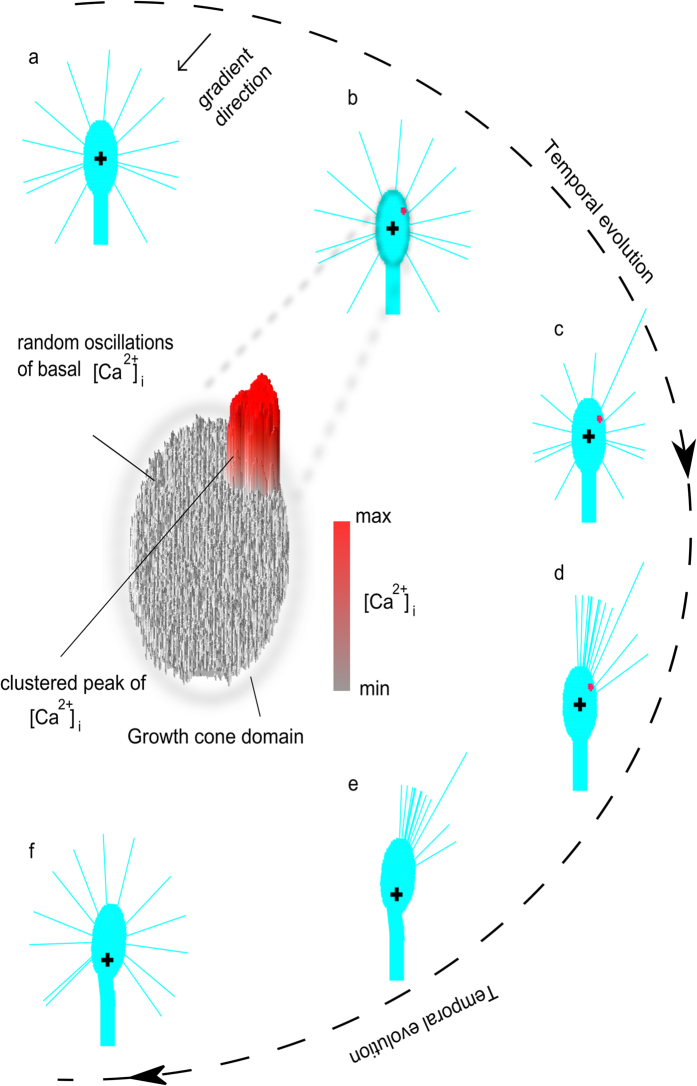
Temporal evolution of the turning response of in silico growth cones. The temporal evolution (external dotted line) of an in silico growth cone exposed to an attractive gradient was shown (the arrow showed the gradient direction, see (**a**)). In particular, a cluster of activator (intracellular calcium) arose within the growth cone side facing the external gradient (**b**). Random oscillations of the basal intracellular calcium together with the calcium peak were shown within the growth cone domain (central magnification of b). (**c**–**f**) The position of the intracellular calcium peak influenced the choice of leading filopodium that guided the growth cone advance. In all CX3D images, the black cross indicates the growth cone barycentre at the beginning of assay.

All simulations were implemented within the multiphysics CX3D framewok. Nevertheless, in standard open code no features accounted for both the presence and the biological action of growth cones. As a consequence, the source code was enriched by creating a novel Java class, and in silico growth cones were implemented as elliptic sets of physical nodes (Fig. 8a). More specifically, physical nodes were geometric points used to identify the vertices of tetrahedrons, deriving from a Delaunay tessellation of the space volume.

To account for biological fluctuation within the growth cone domain, for each node and for each growth step, initial random values of activator (*a*) and inhibitor (*h)* were assigned from uniform distributions. Moreover, to reproduce natural features, several filopodia, extending from the growth cone border with a fan-shaped disposition, were added. To this end, a Java class was written to implement a “filopodium” object. Furthermore, for each growth cone and for each step of growth the number and the angular disposition of filopodia were randomly chosen to reproduce biological variability and stochastic shape fluctuations. The extracellular gradient was implemented as a point source (modelling a single ejection of a chemical solution^52^), and, according to literature^44^, this chemical source was placed 100 μm away from the centre of the growth cone and at a 45 degree angle with respect to y-axis (that is the initial direction of axonal extension). The black arrow in Fig. 8a shows the gradient direction.

Biological experiments accounted for the capability of growth cones to adapt their paths according to the local extracellular environment. Indeed, axon trajectories were found to show several “turning” or “decision” points, where growth cones “update” their outgrowing direction. As a consequence, the in silico outgrowth of axons was modelled as a multistep process, accounting for the whole length of each trajectory (taken from a Gaussian distribution), as well as the number of “decision” points according to experimental trials^44^. In particular, the turning response followed a cyclic behaviour for each outgrowth step. First, intracellular gradients of morphogens (activator (i.e, [Ca^2+^]_i_), and inhibitor) evolved according to Equations (1). This system accounted for random initial conditions and converged to asymmetric patterns, where one or more clusters were polarized towards the chemical source (Fig. 8b). Then, the elongation of filopodia was implemented (Fig. 8c). To this aim, the leading filopodium was chosen to be angularly close to the peak of activator, according to experimental observations, which link local intracellular calcium peaks to the enhanced outgrowth of filopodia^53^. The other simulated filopodia, instead, slowly retracted, while new protrusions sprouted close to the leading filopodium (Fig. 8d). As a consequence, the turning of in silico growth cones was preceded by the preferential protrusion of filopodia on the turning side, according to the biological behaviour^17^,^21^. Thereafter, the whole growth cone simultaneously advanced (Fig. 8e) and turned so that its new orientation was a linear combination between its previous orientation (0.8 times) and the actual angular direction (0.2 times) of the leading filopodium. Finally, filopodia were symmetrically redistributed on the growth cone surface (Fig. 8f).

### Quantitative evaluation of growth cone model performance

Quantitative indexes were used to assess the reliability of the model and its capacity to reproduce the biological behaviour of outgrowing axons. For each in silico growth cone, the displacement of the barycentre was used to trace the axon paths and reduce trajectories to monodimensional lines. As a consequence, the turning angle and the tortuosity of traces were extracted from simulations and compared with the same parameters given by experimental traces to assess their similarity. The turning angle was defined as the angle between the original direction of axon outgrowth and a straight line connecting the positions of growth cone barycentre at the onset and the end of turning assay. Finally, the tortuosity of traces was measured as ratio of curvilinear length of a trajectory to the straight length linking its beginning with its end^37^.

## Additional Information

**How to cite this article**: Morana Roccasalvo, I., Micera, S. & Sergi, P. N. A hybrid computational model to predict chemotactic guidance of growth cones. *Sci. Rep.* **5**, 11340; doi: 10.1038/srep11340 (2015).
